# Increasing dissolved oxygen in water enhances the flooding tolerance of *Carya illinoinensis*

**DOI:** 10.3389/fpls.2026.1755986

**Published:** 2026-02-05

**Authors:** Xue Chen, Xia Wang, Haibo Hu, Chaoming Wu, Li Zhu

**Affiliations:** 1 Co-Innovation Center for Sustainable Forestry in Southern China of Jiangsu Province, Key Laboratory of Soil and Water Conservation and Ecological Restoration of Jiangsu Province, Nanjing Forestry University, Nanjing, China; 2 Department of Resources and Environment, Shandong Water Conservancy Vocational College, Rizhao, China; 3 Wuxi Branch, Bureau of Investigation on Hydrologic Water Resources, Wuxi, China

**Keywords:** *Carya illinoinensis*, dissolved oxygen, flooding, growth, morphology, physiology

## Abstract

Flood-induced seedling mortality along riverbanks is a global issue, primarily caused by oxygen deficiency and associated secondary stresses during submergence. To address this problem, our study introduced an innovative approach by enhancing dissolved oxygen (DO) in floodwater to alleviate flooding stress. We conducted a pot experiment using one-year-old seedlings of two *Carya illinoinensis* cultivars, ‘Mahan’ and ‘Pawnee’, under three treatments: control (CK), high-oxygen flooding (HO), and low-oxygen flooding (LO). Morphological, growth, and physiological responses of both cultivars were systematically evaluated to comprehensively assess their flooding tolerance. The results demonstrated that aeration significantly mitigated both physiological damage and growth inhibition caused by flooding. After 60 days of flooding, compared with the LO treatment, the HO treatment reduced the leaf injury rate (by 11.11% in ‘Mahan’ and 0% in ‘Pawnee’) and the injury index by 26.43–31.75% in both cultivars. It also increased the growth rate in plant height (GRH) by 18.18–166.67%, total biomass (TB) by 15.69–18.17%, and root-to-shoot ratio (RSR) by 18.18–34.94%. Moreover, the HO treatment led to reductions in malondialdehyde (MDA) content by 7.55–7.87%, soluble protein (SP) content by 2.14–20.50%, and activities of superoxide dismutase (SOD) and catalase (CAT) by 16.86–17.16% and 17.20–23.73%, respectively. Membership function analysis further revealed that plants in the HO treatment exhibited superior overall stress resistance compared to those in the LO treatment, with the resistance ranking as follows: ‘Mahan’-HO > ‘Mahan’-LO > ‘Pawnee’-HO > ‘Pawnee’-LO. In summary, this study explores how elevated dissolved oxygen alleviates key flood stress symptoms, thus providing a theoretical foundation for flood-resistant management of *C. illinoinensis* in the Yangtze River Basin and a novel intervention framework for other terrestrial plants facing periodic flooding.

## 1 Introduction

Global warming is associated with an increase in flooding events, which threaten many ecosystems worldwide with submergence (Knapp et al., 2008; Bailey-Serres and Voesenek, 2008). These flooding events represent major abiotic stresses for plants, significantly impacting the global distribution of plant species, community composition, structure, and dynamics (Renziehausen et al., 2024; Li et al., 2020). Research has demonstrated that the primary cause of plant damage under flooding conditions is not the presence of water itself, but rather the hypoxic stress resulting from a drastic decrease in soil oxygen availability (Zeng et al., 2021; Zhao et al., 2021). Oxygen plays an essential role in key physiological processes such as energy metabolism and oxidative phosphorylation, and its availability directly affects plant survival (Zahra et al., 2021). However, oxygen exhibits extremely low solubility in water, with diffusion rates significantly slower than in air (Bailey-Serres and Voesenek, 2008; Du et al., 2016). Consequently, terrestrial plants face severe oxygen deficiency even in water saturated with dissolved oxygen (DO) (Mommer et al., 2004).

Based on their adaptability to water conditions, terrestrial plants can be classified into three major categories: xerophytes, mesophytes, and hydrophytes (Niu et al., 2023). Among these, mesophytes are highly diverse and widely distributed, serving as key components of riparian ecosystems. Unlike aquatic plants adapted to water environments (Mommer et al., 2005), mesophytes grow long-term in oxygen-rich terrestrial environments. Their leaves and stems often possess waxy cuticles, well-developed epidermal layers, and dense cellular structures (Pedersen et al., 2017). However, under flooding conditions, these structures can impede oxygen diffusion into the plant, exacerbating hypoxia (Pedersen et al., 2017). While hypoxic stress impairs essential physiological functions such as photosynthesis (Zhao et al., 2021), energy production (Wang et al., 2025), and nutrient absorption (Iftikhar et al., 2025), ultimately stunting plant growth and reducing yield and quality. To cope with such stress, plants have evolved multiple defense mechanisms against hypoxia over long-term evolution. These strategies include changes in height and ground diameter (Schindler et al., 2020), in photosynthetic product allocation and accumulation (Markus-Michalczyk et al., 2016; Sun L. et al., 2020), and enhancement of antioxidant systems (Zeng et al., 2022), collectively improving their adaptability to hypoxic environments.

Specifically, plant phenotypic traits represent a primary visible response to abiotic stress (Klein et al., 2020). Studies show that hypoxia inhibits new leaf formation, leading to leaf chlorosis and abscission, thereby restricting overall plant growth (Li et al., 2023). Plants may also enhance oxygen balance within the root system through the development of aerenchyma (Phukan et al., 2016). Morphological observations alone are limited to elucidating underlying physiological mechanisms; these must be complemented by the analysis of key chemical and physiological indicators. Malondialdehyde (MDA) content serves as a key indicator of membrane lipid peroxidation, providing a direct measure of oxidative damage to cell membranes (Yu et al., 2015). Furthermore, plants employ osmoregulatory substances and antioxidant enzyme systems that act synergistically to detoxify excess reactive oxygen species and improve stress adaptation (Wang et al., 2015; Hussain et al., 2018). It should be noted that the effectiveness of these adaptive strategies is often significantly modulated by both flooding duration and plant cultivar (Mozo et al., 2021; Zhou et al., 2019).

*Carya illinoinensis* (also known as the American pecan) is a deciduous nut tree of the Juglandaceae family (Briceno-Contreras et al., 2019). In terms of timber value, *C. illinoinensis* is one of the three best hardwood species, along with Juglans nigra and Cerasus maximowiczii. This species is native to Mexico and the United States, but it has been commercially cultivated in several countries, such as Canada, Italy, Brazil, France, Israel, South Africa, Japan, and China (Marco et al., 2021). In China, large-scale demonstration sites for *C. illinoinensis* have been established, particularly in the Yangtze River Delta region (Zhang et al., 2013). However, this region is affected by the high water levels of the Yangtze River, leading to frequent flooding (Zhou et al., 2016). Therefore, it is important to study and protect *C. illinoinensis*, especially to explore ways to enhance its tolerance to low-oxygen environments.

Recent studies have confirmed that *C. illinoinensis* possesses a certain degree of flooding tolerance (Miao et al., 2022; Chen et al., 2025). However, existing research has primarily focused on its physiological responses to hypoxic stress (Li et al., 2023; Basit et al., 2024). It remains unknown whether actively elevating DO concentration in floodwater can enhance this tolerance, and whether such enhancement varies between cultivars. To address this gap, this study implements an environmental regulation strategy centered on actively elevating DO in floodwater. We systematically assessed the efficacy of this intervention on the flooding tolerance of two major *C. illinoinensis* cultivars, ‘Mahan’ and ‘Pawnee’. We hypothesize that: (1) Elevated floodwater DO will mitigate morphological damage (reducing leaf injury rate and index) and growth inhibition; (2) It will alleviate physiological stress by reducing oxidative damage (lower MDA) and modulating the associated adaptive responses in osmoregulation and antioxidant enzymes; and (3) The degree of improvement in overall flooding tolerance conferred by increased DO will exhibit clear cultivar specificity.

## 2 Materials and methods

### 2.1 Plant materials

The experimental materials consisted of one-year-old *C. illinoinensis* seedlings, including two major cultivars from the Yangtze River Delta region: ‘Mahan’ and ‘Pawnee’. All seedlings were sourced from the American Pecan Experimental Base in Zhangmiao Village, Houbai Town, Jurong City, Jiangsu Province. The potted seedling containers were black gallon pots (16 cm upper diameter × 12 cm lower diameter × 30 cm high), each of which was filled with soil up to 2 cm from the upper rim of the pot. The culture medium consisted of garden soil, earthworm soil, and organic fertilizer, which were uniformly mixed at a 3:6:1 ratio. The soil pH was 5.5, the field water capacity was 31.05%, the proportion of organic matter was 2.83%, and the contents of available N, P, and K were 121.25, 51.18, and 93.29 mg kg^−1^, respectively.

### 2.2 Experimental design

In June 2021, uniformly growing (~40 cm high) seedlings were selected and acclimatized for 15 days in the greenhouse with shading function of Nanjing Forestry University’s Xiashu Forestry (N32.07, E119.12). The temperature of the greenhouse ranged from 23.5 to 34.0°C, and the relative humidity ranged from 63.42 to 84.97% throughout the experiment. The experimental design was in randomized blocks in a 3 × 2 factorial scheme, consisting of three treatments (field capacity 75%, high-oxygen flooding, and low-oxygen flooding) and two cultivars (‘Mahan’ and ‘Pawnee’). Each of the six treatment combinations was replicated three times (n = 3), with each replicate consisting of an experimental unit containing three seedlings. Therefore, each treatment combination included 9 seedlings (3 replicates × 3 seedlings), resulting in a total of 54 seedlings.

**Figure 1:**
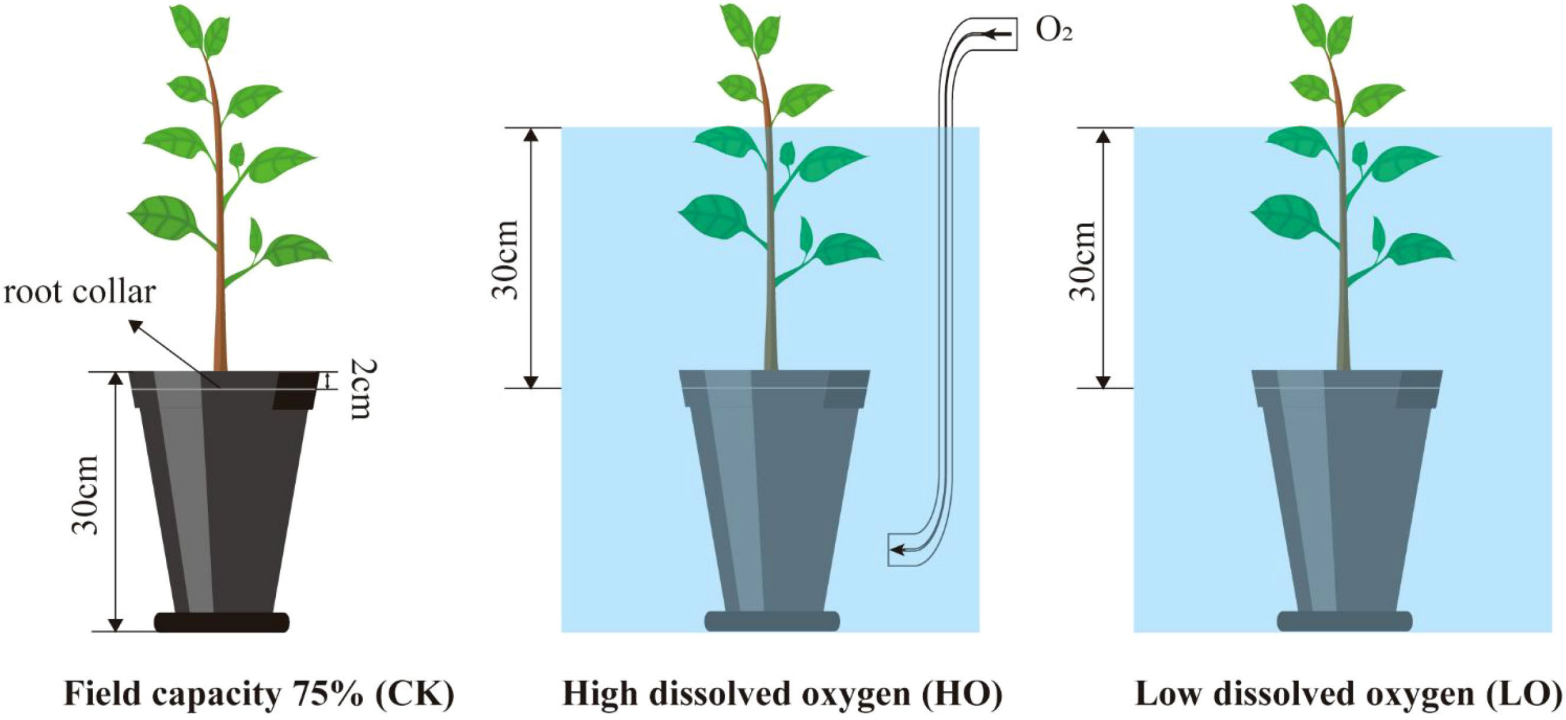
Experimental designs of *C. illinoinensis* to two different dissolved oxygen levels under flooding treatments.

In the period before the beginning of the experiment, all seedlings were watered until percolation commenced, then allowed to drain. Once drainage ceased, indicating that the soil had reached field capacity, each seedling in the control group (CK) was immediately weighed to establish a baseline, and the flooding treatments were initiated. The CK group was maintained at 75% field capacity through daily weighing and supplemental watering. Both the high-oxygen (HO) and low-oxygen (LO) flooding treatments were conducted in low-density polyethylene resin tanks (56 cm × 56 cm × 80 cm), in which the water level was maintained 30 cm above the root collar. No water changes were performed throughout the experiment. In the HO group, continuous aeration was provided using an air pump coupled with nanoporous aeration stones to maintain a high dissolved oxygen environment. In contrast, the LO group relied solely on static flooding via water-air exchange to simulate natural hypoxic flooding conditions (**Figure 1**).

The primary difference between the HO treatment and the LO treatment lies in DO concentrations. The HO treatment maintained an average dissolved oxygen concentration of 7.11 ± 0.10 mg L^-^¹, while the LO treatment averaged 1.88 ± 0.09 mg L^-^¹. All other water quality parameters (temperature, pH, turbidity) remained consistent between treatments, continuously monitored using a Hydrolab multiparameter water quality analyzer (**Table 1**).

**Table 1:** Water environment indicators under two different dissolved oxygen treatments.

Treatments	Dissolved oxygen concentration (mg·L^-1^)	Water temperature (°C)	Water pH	Water turbidity
HO	7.11 ± 0.10^A^	29.37 ± 1.87^A^	8.19 ± 0.11^A^	1.71 ± 0.12^A^
LO	1.88 ± 0.09^B^	29.31 ± 1.88^A^	8.18 ± 0.12^A^	1.74 ± 0.13^A^

Capital letters indicate significant differences among treatment groups (CK, HO, LO) within the same cultivar. Data were expressed as a mean ± SD.

### 2.3 Determination of morphological indicators

Morphological indicators, including leaf numbers, injury numbers, injury grades, injury rate and injury index, were recorded on days 15, 30, 45, and 60, respectively.

The leaf numbers and injury numbers of the seedlings were recorded by manual counting. The classification of injury grades was based on the method described by Zhou et al. (2019) with some adjustments. Grade 1: leaves appeared curling but remained green; Grade 2: leaves showed yellowing; Grade3: leaves showed dark brown patches; Grade 4: leaves were scorched with partial shedding.

Injury rate and injury index were obtained from Equation 1, 2, respectively:

(1) $$\text{Injury rate}=\frac{\text{number of injured seedlings}}{\text{total number of seedlings}}$$

(2) $$\text{Injury Index}=\frac{\sum \left(\text{injury grade}\times \text{number of injured seedlings}\right)}{\text{total number of seedlings}\times \text{maximum injury grade}}\times 100\%$$

Calculation example: On the 60th day of flooding, for the ‘Mahan’ cultivar under the HO treatment, a sample of 9 seedlings was assessed: 4 seedlings were classified as Grade 1, 2 as Grade 2, 1 as Grade 3, and 1 as Grade 4. Therefore, the maximum injury grade was 4. The injury index was then calculated as follows: Injury Index= [(1×4)+(2×2)+(3×1)+(4×1)]/(9×4) = 0.417.

### 2.4 Determination of growth indicators

The seedling height was determined with a measuring tape, while the ground diameter was measured using vernier calipers. Akin to the morphological indicators, the heights and ground diameters of the seedlings were recorded on days 0, 15, 30, 45, and 60, respectively. Thus, the growth rates of height and ground diameter were calculated according to Equation 3:

(3) $$\text{R}=\frac{{\text{Y}}_{2}-{\text{Y}}_{1}}{{\text{X}}_{2}-{\text{X}}_{1}}=\frac{\Delta \text{Y}}{\Delta \text{X}}$$

where, R is the rate of height or ground diameter; Y is the amount of change in the mean value of height or ground diameter; and X is the corresponding time variation.

All seedlings were extracted to determine biomass at the conclusion of the experiment (day 60). For the dry mass quantification, *Caray illinoinensis* seedlings were dug out whole and divided into three portions (roots, stems, and leaves) and then washed and dried at 80 °C to a constant mass, whereafter the dry masses of the three portions were determined separately. The root-to-shoot ratio (RSR) was obtained using Equation 4:

(4) $$\text{Root to shoot ratio}=\frac{\text{root biomass}}{\text{aboveground biomass}}$$

### 2.5 Determination of physiological and biochemical indicators

Mature leaves from the middle sections of the seedlings were harvested on days 15, 30, 45, and 60. Some leaves were taken for the determination of plasma membrane permeability, and the rest were stored in ultra-low temperature refrigerator (-80 °C) for the determination of other physiological indicators.

The malondialdehyde (MDA) content was extracted using a thiobarbituric acid (TBA) reagent and boiled at 100 °C for 20 min according to the method of Wassie et al. (2019). Once the solution was cooled to room temperature, it was centrifuged at 3000 rpm for 10 min. The absorbance of the supernatant was then measured using a spectrophotometer (Shimadzu UV-2450, Shimadzu Corporation, Kyoto, Japan) at 450, 532, and 600 nm. The MDA concentration was estimated using Equation 5:

(5) $$\text{MDA }\left(\text{μmol}/\text{g FW}\right)=6.45\left(A_{532}-A_{600}\right)-0.56A_{450}$$

where A_450_, A_532_, and A_600_ are the absorbance of the supernatant at 450, 532, and 600 nm, respectively.

The soluble protein (SP) content was determined via a Coomassie brilliant blue G-250 staining method. A 0.1 g volume of leaves was ground using a mortar with a 1 mL phosphate buffer solution (PBS, pH = 7.8), and then transferred to a 10 mL centrifuge tube. The material was then washed twice with 2 mL PBS and transferred to a 10 mL centrifuge tube and centrifuged at 10,000 rpm for 15 min at 4 °C. After centrifugation, the 0.1 mL supernatant was transferred into a test tube, to which 5 mL of Coomassie brilliant blue G-250 was added and thoroughly mixed. Two min later, the absorbance of the resulting solution was measured at 595 nm using a spectrophotometer. The soluble protein was calculated according to Equation 6:

(6) $$\text{Soluble protein }\left(\text{mg}/\text{g FW}\right)=\frac{\text{C}\times {\text{V}}_{T}}{\text{W}\times {\text{V}}_{S}\times 1000}$$

where C is the protein content from a standard curve; V_T_ is the total volume of extracting solution; W is the sample weight; and V_S_ is the sampling volume of determination.

The superoxide dismutase (SOD) activity was measured through a nitro blue tetrazolium (NBT) reduction method. To prepare the enzyme solution: 0.5 g of leaves were ground into a homogenate with a 10 mL of phosphate buffer (PBS, pH = 7.8), transferred to a centrifuge tube, and centrifuged at 12,000 rpm for 20 min at 4 °C. The supernatant was a SOD crude enzyme solution. The reaction solution consisted of a 14.5 mM methionine (Met) solution, 3 mM ethylenediaminetetraacetic acid disodium salt (EDTA-Na2) solution, 60 μM riboflavin solution, and 2.25 mM NBT solution. Subsequently, 2.9 mL of the reaction mixture and 0.1 mL of the enzyme solution were extracted via testing tubes. Two control tubes were prepared simultaneously, one with 2.9 mL of a reaction mixture and 0.1 mL of PBS (without the enzyme solution) as the maximum light reduction tube, and the other with 2.9 mL of the reaction mixture and 0.1 mL of PBS while wrapped in tin foil to block the light. The tubes were then placed in a light incubator at 4000 lux for 20 min at 25 °C and then in darkness to stop the reaction. Following the reaction, the spectrophotometer was zeroed with the control tube without illumination, and the absorbance of each tube was measured separately at 560 nm. One unit of SOD activity was defined as the amount of enzyme that inhibited the NBT reduction by 50%. The SOD activity was calculated according to Equation 7:

(7) $$\text{SOD }\left(\text{U}/\text{g FW}\right)=\frac{({\text{A}}_{0}-{\text{A}}_{\text{S}})\times V_{T}}{0.5\times {\text{A}}_{0}\times \text{W}\times V_{1}}$$

where A_0_ is the absorbance of control; A_S_ is the absorbance of the sample; V_T_ is the total volume of the extracting solution; W is the sample weight; and V_1_ is the sampling volume of determination.

The catalase (CAT) activity was determined using a UV absorption method. For enzyme extraction, 0.5 g of leaves was weighed in a mortar, and 2–3 mL of a pre-cooled phosphate buffer (PBS, pH = 7.8) was added at 4 °C with a small amount of quartz sand and ground into a homogenate. This material was transferred to a 25 mL volumetric flask, and the mortar was rinsed several times with buffer solution to fix the volume on the scale. Subsequently, the volumetric flask was placed in a refrigerator at 5 °C for 10 min, and the upper clarified liquid was centrifuged at 4000 rpm for 15 min. The supernatant was the CAT crude enzyme. For the determination of enzyme activity, three 10 mL test tubes were used (two for sample measurement and one as a blank) and designated as S0, S1, and S2 respectively, to which 0.2 mL of the crude enzyme solution, 1.5 mL of a phosphate buffer (PBS, pH = 7.8), and 1.0 mL of distilled water were added, respectively. The test tube S0 was heated in a boiling water bath for 1 min to deactivate the enzyme solution and cooled. All of the test tubes were then preheated at 25 °C to which 0.3 mL of 0.1 mol/L H_2_O_2_ was added one by one. Each tube was quickly timed and quickly poured into a quartz cuvette, and the absorbance was measured at 240 nm with a 1 min reading at each interval for 4 min. Once all three tubes were measured, the enzyme activity was calculated. A unit of CAT activity was defined as the changes in absorbance at 240 nm per min. The CAT activity was calculated using Equations 8 and 9:

(8) $$\text{CAT }\left(\text{U}/\text{gFW}\right)=\frac{{\text{ΔA}}_{240}\times {\text{V}}_{T}}{0.1\times {\text{V}}_{S}\times \text{t}\times \text{W}}$$

(9) $$\text{Δ}A_{240}={\text{A}}_{S0}-\frac{\left({\text{A}}_{S1}+{\text{A}}_{S2}\right)}{2}$$

where ΔA240 is the absorbance (control minus the assay); V_T_ is the total volume of extracting solution; V_S_ is the sampling volume of determination; t is the time from the beginning of the addition of H_2_O_2_ to the last reading; and W is the sample weight.

### 2.6 Statistical analysis

The experimental data were subjected to preliminary statistical analysis and multivariate analysis of variance (MANOVA) for between-subject effects using SPSS 20.0 software. *Post hoc* comparisons were carried out with Tukey’s HSD test at a 95% confidence level, with statistical significance defined as p< 0.05. The data were expressed as mean ± standard deviation (SD).

Comprehensive evaluation of flooding stress tolerance was performed using the membership function method described by Liang et al. (2025). This analysis was based on the key morphological, growth, and physiological indicators measured after 60 days of flooding stress. To eliminate the influence of inherent trait differences between cultivars, the resistance coefficient (X) for each indicator was first calculated separately for each cultivar using Equation 10:

(10) $$\text{X}=\frac{\text{Measured value under flooding stress}}{\text{Measured value in CK}}$$

The membership function value (U) was then calculated for each indicator of each cultivar. The average of these values (D) was used for comparison, where a higher D value indicates stronger comprehensive stress resistance of the cultivar.

(11) $$\text{U }\left({\text{X}}_{ij}\right)=\frac{{\text{X}}_{ij}-{\text{X}}_{j\min}}{{\text{X}}_{j\max}-{\text{X}}_{jmin}}$$

(12) $$\text{U }\left({\text{X}}_{ij}\right)=1-\frac{{\text{X}}_{ij}-{\text{X}}_{j\min}}{{\text{X}}_{j\max}-{\text{X}}_{jmin}}$$

Where X_ij_ denotes the measured value of indicator j for cultivar i, while X_jmax_ and X_jmin_ represent the maximum and minimum values of indicator j across all cultivars, respectively. Equation 11 was applied when indicator j was positively correlated with stress resistance; otherwise, Equation 12 was used.

The comprehensive evaluation value (D-value) for each cultivar under each treatment was calculated as the mean of all membership function values (U) according to Equation 13:

(13) $$\text{D}=\frac{1}{\text{n}}\sum_{j=1}^{n}\text{U}({\text{X}}_{ij})$$

where n is the number of indicators included in the analysis. A higher D-value indicates stronger overall flooding stress tolerance. The D-values were finally used to rank all treatment–cultivar combinations.

## 3 Results

### 3.1 Morphology indicators of *C. illinoinensis* under different treatments

#### 3.1.1 Injury numbers and injury grades

**Figure 2:**
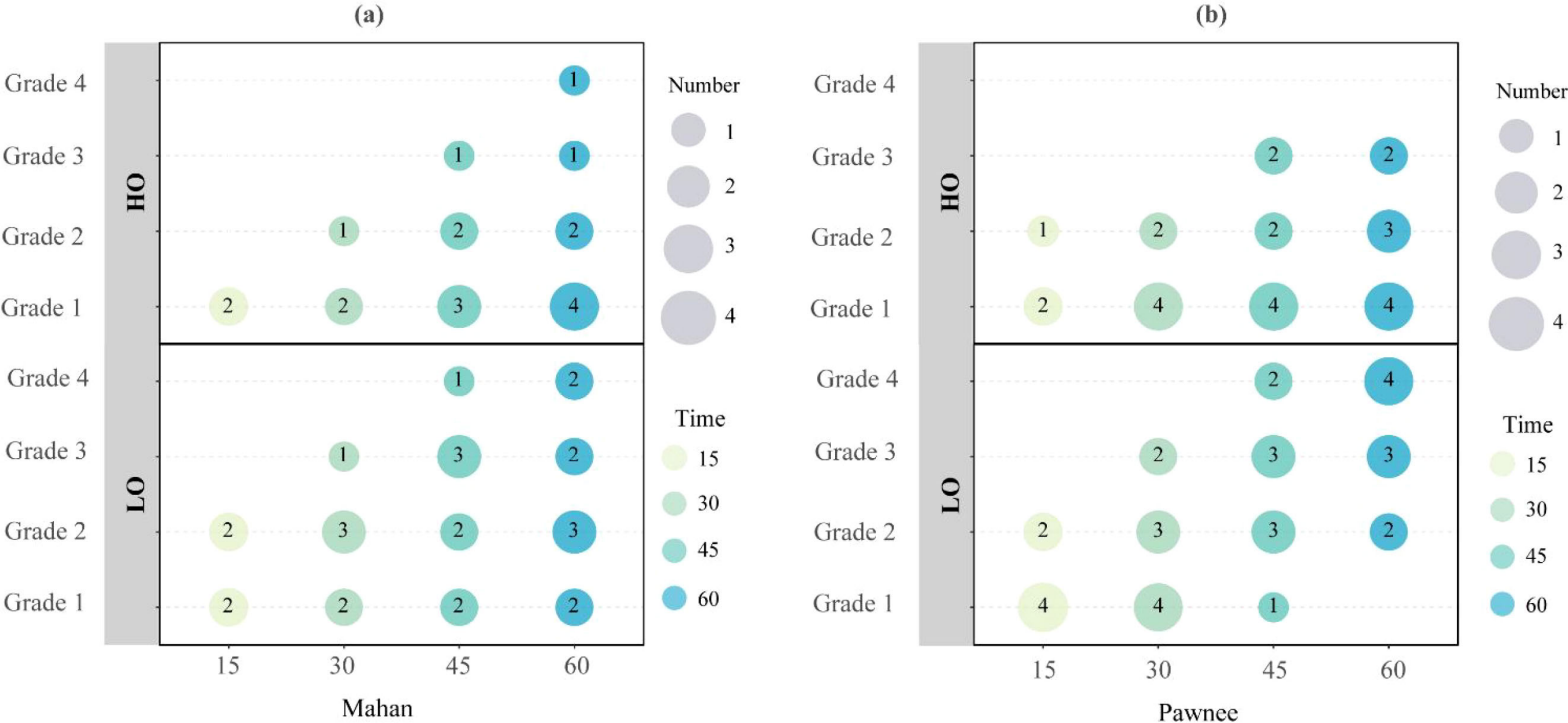
Injury number and injury grade of ‘Mahan’ **(a)** and ‘Pawnee’ **(b)** cultivars under different treatments during the experimental period. Circle color indicates the sampling day (15, 30, 45, 60 days), and circle size represents the number of injured plants.

For the ‘Mahan’ cultivar under HO treatment, the injury number gradually increased from 2 (all Grade 1) on day 15 to 8 (Grades 1–4) by day 60 (**Figure 2a**). In contrast, under LO treatment, injury was more severe, with 4 plants affected (Grades 1–2) as early as day 15, rising to 9 plants (Grades 1–4) by day 60 (**Figure 2a**).

The ‘Pawnee’ cultivar exhibited a similar trend but overall greater injury severity than ‘Mahan’ (**Figure 2b**). Under HO treatment, its injury number increased from 3 (Grades 1–2) on day 15 to 9 (Grades 1–3) on day 60. Under LO treatment, 6 plants were already injured (Grades 1–2) by day 15, and the number reached 9 (Grades 2–4) by day 60 (**Figure 2b**).

In comparison, the CK groups of both cultivars exhibited robust leaf growth throughout the trial period with no observed damage symptoms. Consequently, both the injury number and the injury grade were zero. Throughout the experimental period (within 60 days), both cultivars consistently showed a higher injury number under LO treatment than under HO treatment. And plants with higher injury grades (Grades 3–4) appeared earlier and accounted for a larger proportion under LO treatments.

#### 3.1.2 Injury rate and injury index

Based on the injury number, injury grade, and total number of seedlings, the injury rate and injury index were calculated to reflect the extent and intensity of flooding damage, respectively (**Table 2**). The injury rate of ‘Mahan’ continuously increased throughout the experiment under both HO and LO treatments, with values in the HO group remaining consistently lower than those in the LO group. In contrast, the injury rate of ‘Pawnee’ increased more rapidly, reaching 100% by day60 under HO treatment and as early as day30 under LO treatment.

**Table 2:** Injury rate and injury index of the two *C. illinoinensis* cultivars under high-oxygen (HO) and low-oxygen (LO) flooding treatments.

Cultivar	Treatment	Injury rate (%)				Injury index			
		15	30	45	60	15	30	45	60
Mahan	HO	22.22	33.33	66.67	88.89	0.222	0.222	0.370	0.417
	LO	44.44	66.67	88.89	100	0.333	0.407	0.528	0.611
Pawnee	HO	33.33	66.67	88.89	100	0.407	0.444	0.519	0.593
	LO	66.67	100	100	100	0.444	0.593	0.667	0.806

A similar trend was observed in the injury index. With the exception of ‘Mahan’ under HO treatment, which maintained an identical injury index (0.222) on days15 and30, all other treatment combinations exhibited a progressive increase in the injury index over time. Furthermore, both cultivars consistently showed lower injury index values under HO treatment compared to the LO treatment. Overall, under the same treatment conditions, ‘Pawnee’ exhibited higher injury rates and injury indices than ‘Mahan’ throughout the experimental period.

### 3.2 Growth indicators of *C. illinoinensis* under different treatments

#### 3.2.1 Height and ground diameter

Regarding the growth rate in plant height (GRH), the three-way interaction among cultivar, time, and treatment was not significant (**Table 3**). However, all three main factors (cultivar, time, and treatment) and their two-way interactions had a significant influence on GRH (p<0.05). For the growth rate in ground diameter (GRD), the main effects of cultivar, time, and treatment, as well as all their interactions, exhibited highly significant effects (p<0.001, **Table 3**).

**Table 3:** Analysis of variance on between-subject effects.

Source of variation		Cultivar	Time	Treatment	Cultivar×Time	Cultivar×Treatment	Time×Treatment	Cultivar×Time×Treatment
GRH	F	8.555^**^	12.576^***^	436.077^***^	3.187^*^	9.399^***^	2.636^*^	0.664
GRD	F	372.001^***^	149.308^***^	1840.169^***^	72.249^***^	87.217^***^	50.050^***^	23.268^***^
TB	F	0.001	/	14.088^***^	/	0.102	/	/
RSR	F	5.692^*^	/	18.058^***^	/	1.966	/	/
MDA	F	273.279^***^	165.919^***^	388.770^***^	22.499^***^	13.541^***^	12.219^***^	5.787^***^
SP	F	2796.360^***^	413.894^***^	5098.298^***^	299.422^***^	88.064^***^	52.298^***^	38.025^***^
SOD	F	162.996^***^	232.321^***^	2105.107^***^	6.607^***^	12.620^***^	48.967^***^	3.787^**^
CAT	F	1358.744^***^	220.235^***^	3912.770^***^	1.065	106.704^***^	64.535^***^	3.089^**^

*represents *p* < 0.05, **represents *p* < 0.01, and ***represents *p* < 0.001. GRH, represents growth rate in plant height; GRD, represents growth rate in ground diameter; TB, represents total biomass; RSR, represents root to shoot ratio; MDA, represents malondialdehyde; SP, represents soluble protein; SOD, represents superoxide dismutase; CAT represents catalase.

**Figure 3:**
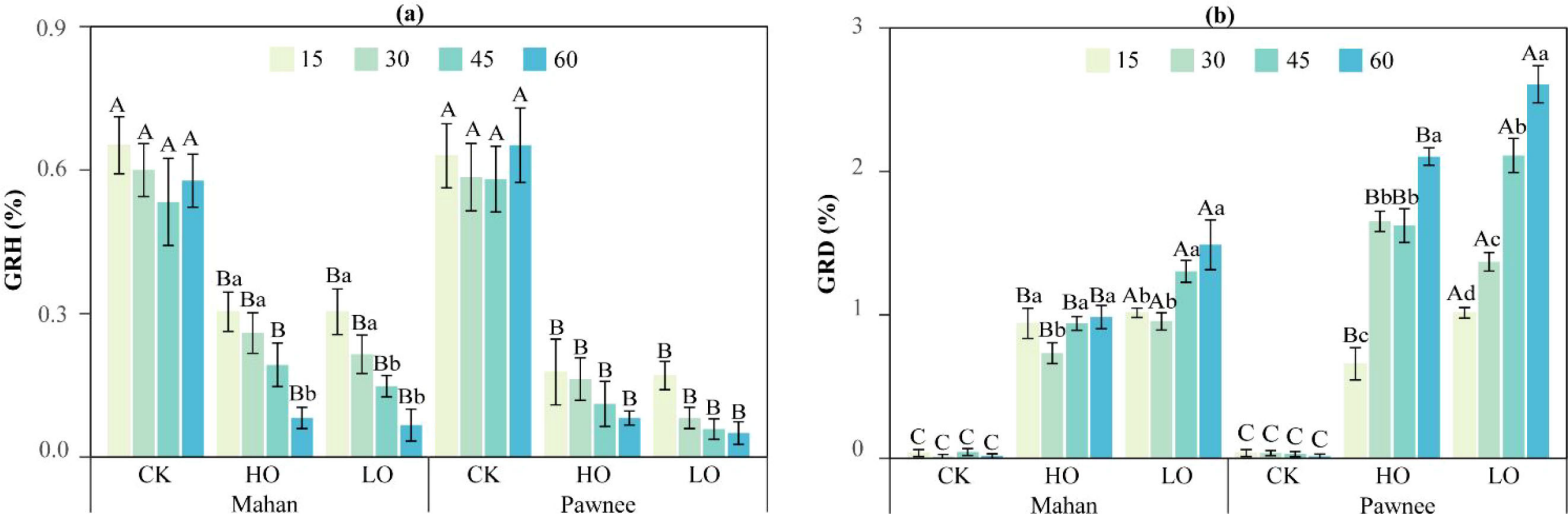
Changes in plant height **(a)** and ground diameter **(b)** of the two *C. illinoinensis* cultivars under different treatments (CK, HO, LO) during the experimental period. Capital letters indicate significant differences among treatments (CK, HO, LO), and lowercase letters indicate significant differences among sampling times (15, 30, 45, and 60 days). Data were expressed as a mean ± SD.

As the flooding duration extended, both the ‘Mahan’ and ‘Pawnee’ cultivars showed a continuous decline in GRH under HO and LO treatments (**Figure 3a**). Specifically, for ‘Mahan’, GRH decreased by 53.41%–85.90% across all time under HO treatment and by 53.41%–88.46% under LO treatment relative to CK. Similarly, ‘Pawnee’ exhibited a marked decreasing trend, with GRH reductions ranging from 71.76%–94.32% under HO and 72.94%–94.32% under LO conditions compared to CK. These results indicate that both HO and LO treatments significantly inhibited seedling height growth.

For GRD, both cultivars exhibited significantly higher values under HO and LO flooding treatments compared to CK, and the LO treatment consistently resulted in greater GRD than the HO treatment (**Figure 3b**). Specifically, ‘Mahan’ reached its maximum GRD during the 45–60 days period (HO: 0.15 mm; LO: 0.22 mm), while its minimum values were recorded between 15–30 days (HO: 0.11 mm; LO: 0.14 mm). Similarly, ‘Pawnee’ achieved its maximum GRD at 45–60 days (HO: 0.32 mm; LO: 0.39 mm), with the minimum occurring within the first 0–15 days (HO: 0.10 mm; LO: 0.15 mm).

Overall, throughout the 60-day experimental period, the plant height growth rate of both cultivars followed the order: CK > HO > LO (**Table 4**). Specifically, under CK conditions, ‘Pawnee’ exhibited a higher GRH than ‘Mahan’, whereas under both HO and LO flooding treatments, ‘Mahan’ showed superior GRH. In contrast, the GRD for both cultivars generally adhered to the pattern of CK< HO< LO. Under these flooding treatments, ‘Pawnee’ demonstrated a greater GRD than ‘Mahan’, highlighting cultivar-specific responses to the treatments.

**Table 4:** Plant height and ground diameter of the two *C. illinoinensis* cultivars under different treatments at day 60.

Cultivar	Treatment	GRH (%)	GRD (%)
Mahan	CK	0.59 ± 0.09^A^	0.02 ± 0.01^C^
	HO	0.21 ± 0.06^B^	0.90 ± 0.04^B^
	LO	0.18 ± 0.05^B^	1.19 ± 0.08^A^
Pawnee	CK	0.64 ± 0.08^A^	0.03 ± 0.01^C^
	HO	0.13 ± 0.04^B^	1.51 ± 0.13^B^
	LO	0.05 ± 0.03^B^	1.78 ± 0.08^A^

Capital letters indicate significant differences among treatment groups (CK, HO, LO) within the same cultivar. Data were expressed as a mean ± SD.

#### 3.2.2 Total biomass and root to shoot ratio

There were significant differences in total biomass (TB) among the treatments (*p* < 0.001). Similarly, root-to-shoot ratio (RSR) differed significantly among treatments (*p* < 0.001), and also between cultivars (*p* < 0.05). The treatment × cultivar interaction was not significant for either TB or RSR (**Table 3**).

**Figure 4:**
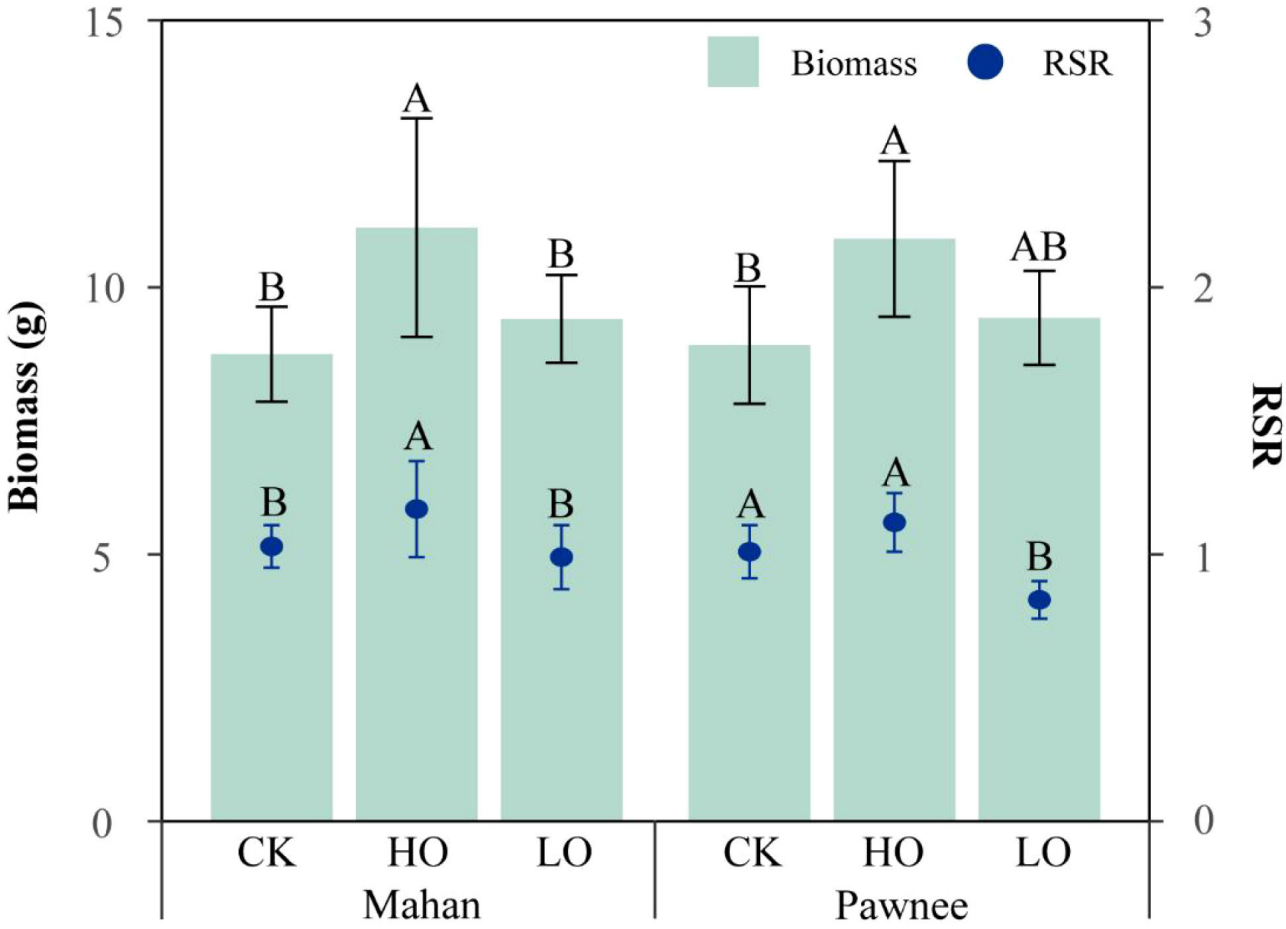
Total biomass and root-to-shoot ratio of two *C. illinoinensis* cultivars under different treatments (CK, HO, LO) at the end of the experiment (day 60). Capital letters indicate significant differences among treatments (CK, HO, LO). Data were expressed as a mean ± SD.

At harvest (day 60), a significant growth-promoting effect was observed under HO treatment for both cultivars (**Figure 4**). Specifically, for ‘Mahan’, TB and RSR under HO treatment reached 11.12 g and 1.17, respectively, which were significantly higher than those in both the CK and LO treatments. For ‘Pawnee’, TB under HO treatment increased by 22.31% compared to the CK. The highest RSR for this cultivar was also observed under HO treatment (1.12), followed by CK (1.01), with the LO treatment yielding the lowest value (0.83).

### 3.3 Physiological indicators of *C. illinoinensis* under different treatments

#### 3.3.1 Malondialdhyde

**Figure 5:**
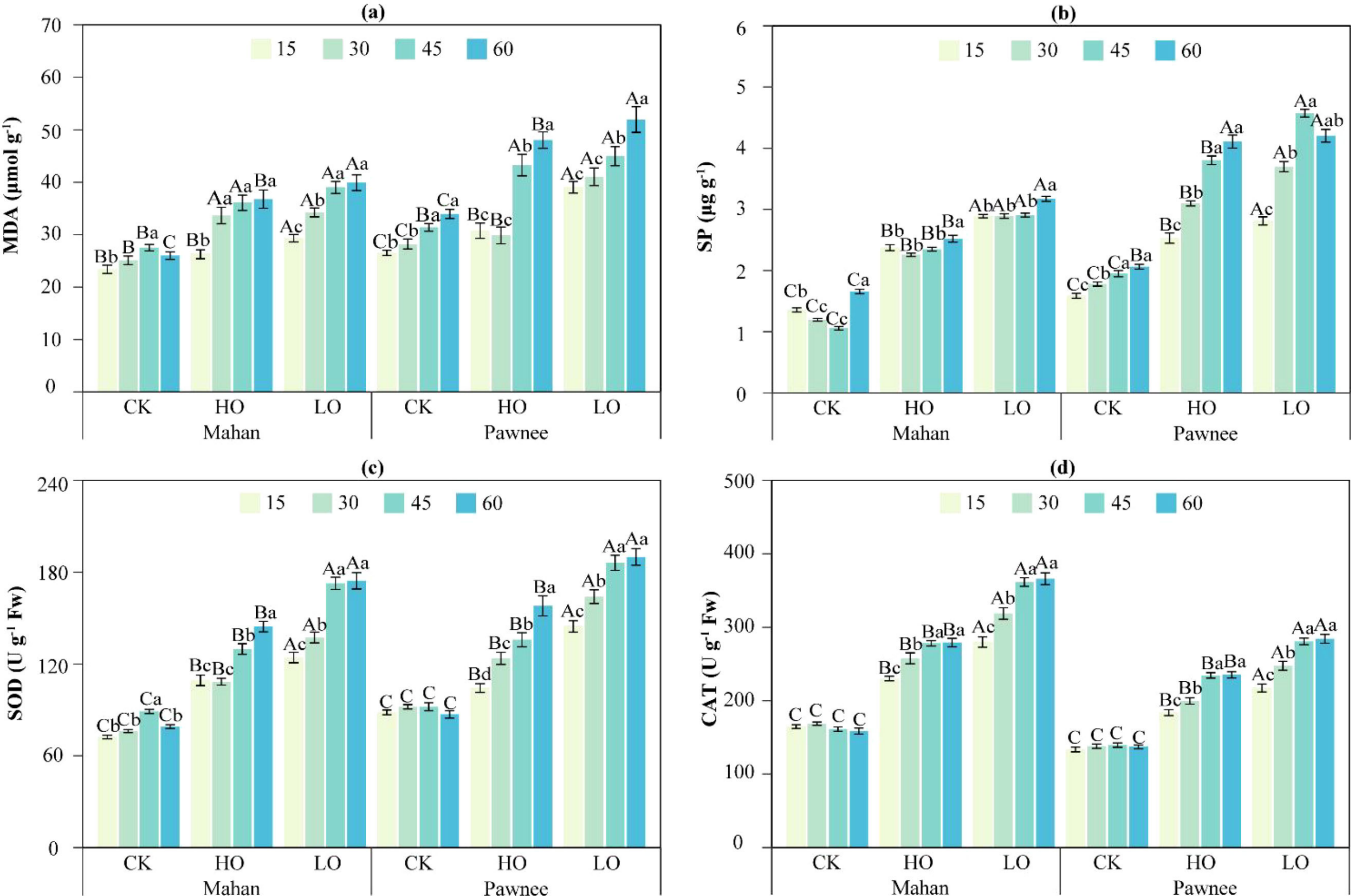
The Malondialdhyde [MDA, **(a)**], Soluble Protein [SP, **(b)**], Superoxide Dismutase [SOD, **(c)**], and Catalase [CAT, **(d)**] in two *C. illinoinensis* cultivars under different treatments (CK, HO, LO) during the experimental period. Capital letters indicate significant differences among treatments (CK, HO, LO), and lowercase letters indicate significant differences among sampling times (15, 30, 45, and 60 days). Data were expressed as a mean ± SD.

MDA content was significantly influenced by cultivar, time, treatment, and all their interactions (*p* < 0.001, **Table 3**). As shown in **Figure 5a**, the MDA content in both ‘Mahan’ and ‘Pawnee’ cultivars increased over time under HO and LO treatments, reaching peak values at day 60 (‘Mahan’: 36.77–39.91 μmol·g^-^¹; ‘Pawnee’: 48.02–51.94 μmol·g^-^¹).

In ‘Mahan’, MDA content under LO treatment was significantly higher than those in CK by 25.35%–53.43% across all time. Under HO treatment, MDA content was also significantly elevated compared to CK at 30 and 45 days, with increases of 33.99% and 31.10%, respectively. Notably, at 15 and 60 days, MDA content in the HO treatment was significantly lower than those in LO treatment, showing reductions of 10.44% and 7.86%, respectively.

A similar pattern was observed in ‘Pawnee’. Both LO and HO treatments resulted in significantly higher MDA content than CK throughout the experiment, with increases ranging from 43.29%–47.16% and 5.84%–41.48%, respectively. Furthermore, at 15, 30, and 60 days, the MDA content under HO treatment was significantly lower than that under LO treatment, with reductions of 21.21%, 27.24%, and 7.55%, respectively.

#### 3.3.2 Soluble protein

Significant effects on soluble protein (SP) were observed for cultivar, time, treatment, and their interactions (*p* < 0.001, **Table 3**). As shown in **Figure 5b**, the ‘Mahan’ cultivar reached its peak SP content at 60 days under both HO and LO treatments (HO: 2.52 μg·g^-^¹; LO: 3.17 μg·g^-^¹). In contrast, the ‘Pawnee’ cultivar showed different peak timing: under HO treatment, SP peaked at 60 days (4.11 μg·g^-^¹), whereas under LO treatment, it peaked earlier at 45 days (4.57 μg·g^-^¹).

Throughout the experiment, the ‘Mahan’ cultivar maintained its highest SP content under LO treatment (2.89–3.17 μg·g^-^¹), followed by HO treatment (2.26–2.52 μg·g^-^¹), with the CK showing the lowest values (1.06–1.66 μg·g^-^¹). For the ‘Pawnee’ cultivar, both LO and HO treatments resulted in significantly higher SP levels than the CK, with increases of 77.30%–134.47% and 59.56%–99.24%, respectively. Additionally, at 15, 30, and 45 days, the HO treatment yielded significantly lower SP content than the LO treatment in ‘Pawnee’, with reductions of 9.96%, 16.22%, and 16.85%, respectively.

#### 3.3.3 Superoxide dismutase

Analysis of variance indicated that cultivar, time, treatment, and their interactions significantly influenced SOD activity, with the three-way interaction significant at P< 0.01 and all other effects at *P* < 0.001 (**Table 3**). As shown in **Figure 5c**, SOD activity in both the ‘Mahan’ and ‘Pawnee’ cultivars peaked at 60 days under HO and LO treatments. For ‘Mahan’, peak SOD activities under HO and LO treatments reached 144.48 U·g^-^¹ FW and 174.41 U·g^-^¹ FW, respectively, representing increases of 82.79% and 120.65% compared to the CK. Notably, the HO treatment showed a significant reduction of 17.16% compared to the LO treatment. A similar trend was observed in ‘Pawnee’, where peak activities under HO and LO treatments were 157.99 U·g^-^¹ FW and 190.03 U·g^-^¹ FW, respectively, corresponding to increases of 81.42% and 118.21% over the CK. In this cultivar, the HO treatment also resulted in a significant decrease of 16.86% relative to the LO treatment.

#### 3.3.4 Catalase

Analysis of variance revealed that all main effects and interactions on CAT activity reached significance (*p* < 0.01 or *p<* 0.001), with the exception of the cultivar × time interaction, which was not significant (**Table 3**). As shown in **Figure 5d**, CAT activity in both the ‘Mahan’ and ‘Pawnee’ cultivars peaked at 60 days under HO and LO treatments. For the ‘Mahan’ cultivar, peak CAT activities under HO and LO treatments reached 279.26 U·g^-^¹ FW and 366.16 U·g^-^¹ FW, respectively, corresponding to increases of 76.01% and 130.78% compared to the CK. The HO treatment resulted in a significant reduction of 23.73% relative to the LO treatment. A similar trend was observed in the ‘Pawnee’ cultivar, where peak activities under HO and LO treatments were 235.40 U·g^-^¹ FW and 284.29 U·g^-^¹ FW, respectively, representing increases of 71.59% and 107.22% over the CK. In this cultivar, the HO treatment also led to a significant decrease of 17.20% compared to the LO treatment.

### 3.4 Stress tolerance analysis and evaluation

The comprehensive stress tolerance of the two cultivars under different flooding treatments was evaluated using the membership function method. As summarized in **Table 5**, the average membership function value (D-value) for ‘Mahan’ under HO and LO treatments was 0.56 and 0.47, respectively, while that for ‘Pawnee’ was 0.43 and 0.35. Based on these D-values, the stress resistance of the treatment–cultivar combinations followed the rank order: ‘Mahan’–HO > ‘Mahan’–LO > ‘Pawnee’–HO > ‘Pawnee’–LO. (**Table 5**).

**Table 5:** Membership function values and ranking of different indexes of different varieties.

Cultivar	Treatment	U(Xij)										Mean	Ranking
		IR	II	GRH	GRD	TB	RSR	MDA	SP	SOD	CAT		
Mahan	HO	1.000	1.000	0.608	0.764	0.612	0.452	0.618	0.002	0.301	0.255	0.561	1
	LO	0.000	0.501	0.521	0.593	0.185	0.243	0.443	0.607	0.726	0.838	0.466	2
Pawnee	HO	0.000	0.548	0.319	0.683	0.507	0.421	0.616	0.727	0.286	0.208	0.431	3
	LO	0.000	0.000	0.162	0.576	0.145	0.066	0.449	0.797	0.699	0.587	0.348	4

IR, represents injury rate; II, represents injury index; GRH, represents growth rate in plant height; GRD, represents growth rate in ground diameter; TB, represents total biomass; RSR, represents root to shoot ratio; MDA, represents malondialdehyde; SP, represents soluble protein; SOD, represents superoxide dismutase; CAT, represents catalase.

## 4 Discussion

The frequency of extreme flooding events under future climate change scenarios, particularly in the Yangtze River Basin, poses a significant threat to the growth of neighboring forests and presents a serious challenge to natural resource managers (He et al., 2022; Yang et al., 2021). The principal flood season in this region occurs annually from July to August (Qian et al., 2021). As an important economic tree species in the area, *C. illinoinensis* is frequently subjected to flooding stress during this period, which significantly compromises its yield and quality. Consequently, improving the flooding tolerance of this species has emerged as a key issue in contemporary forestry management. This study investigates whether increasing DO concentrations in floodwater can enhance the flooding tolerance of *C. illinoinensis*, with the aim of providing theoretical support for ecological management of the Yangtze River Basin Shelterbelt System. To account for potential intraspecific variation, two major cultivars, ‘Mahan’ and ‘Pawnee’, were selected for systematic comparison of their morphological, growth, and physiological responses under flooding conditions.

### 4.1 Morphology

Morphological structure serves as a direct indicator of plant damage under abiotic stress (Zahedi et al., 2025; Fang et al., 2025). Under flooding conditions, plants often develop symptoms such as leaf yellowing, desiccation, and abscission (Bispo and Vieira, 2022; Wiström et al., 2023). To systematically evaluate the degree of leaf damage across different treatments, this study quantitatively analyzed injury rate and injury index. Results showed that compared to CK, both cultivars demonstrated increased injury indicators (IR and II) under HO and LO treatments (**Table 2**). This is similar to the results reported by Zhang et al. (2025), which indicate that flooding induces root hypoxia, inhibits photosynthesis, and drives plants toward anaerobic respiration. Anaerobic respiration not only leads to insufficient energy supply but also accumulates toxic substances such as alcohol, lactic acid, and acetaldehyde, which are harmful to plant. Notably, under the HO treatment, both cultivars exhibited lower injury indicators than under LO treatment (**Table 2**), suggesting that increasing environmental DO effectively mitigates leaf damage caused by flooding (Du et al., 2016). This may be attributed to the oxygenation treatment directly improving oxygen levels in the root zone, delaying metabolic disorders caused by anaerobic respiration, and maintaining normal root physiological functions, thereby enhancing seedling tolerance to flooding (Li et al., 2025). Furthermore, we observed differences in injury indicators between cultivars (**Table 2**). The ‘Mahan’ cultivar exhibited a slower increase in injury rate under both treatments, reflecting stronger stress tolerance. In contrast, the ‘Pawnee’ cultivar not only showed a more rapid rise in injury rate but also reached 100% injury earlier under LO conditions, indicating higher sensitivity to root-zone hypoxia.

### 4.2 Growth

This study demonstrates that both HO and LO treatments significantly inhibited GRH in both cultivars (**Table 4**). This observation is consistent with the Low Oxygen Quiescence Syndrome (LOQS) strategy proposed by Voesenek and Bailey-Serres (2015), in which plants actively slow their growth under flooding to minimize energy consumption and prolong survival under stress (Fan et al., 2018; Sun H, et al., 2020). Notably, compared to the LO treatment, the HO treatment exhibited a reduced inhibitory effect on plant height growth (**Table 4**). This indicates that increasing DO levels can partially alleviate the suppression of plant height elongation caused by flooding stress. Similar phenomena have been reported in tomatoes, where supplemental aeration significantly improved plant height growth oxygenation treatments effectively promote plant height growth under hypoxic conditions (Zheng et al., 2017). In contrast, both cultivars showed a stimulatory effect on GRD under flooding, with values following the order CK< HO< LO (**Table 4**). This morphological response can be attributed to the formation of hypertrophic lenticels at the stem base. Research indicates that hypertrophic lenticels is the signature morphological response of flood-tolerant woody plants to flooding (Phukan et al., 2016; Argus et al., 2015). Plants adapted by increasing the total area of O2-absorbing tissues exposed to the air (Li et al., 2023), thereby enhancing the ventilation of flooded organs to avoid energy crises caused by hypoxia (Fukao et al., 2019).

The allocation of biomass between aboveground and belowground parts represents an important adaptive strategy of plants to abiotic stress (Poorter et al., 2012). In this study, the ‘Pawnee’ cultivar exhibited a significantly lower RSR under LO treatment than under the CK conditions (**Figure 4**), reflecting a marked reduction in the proportion of photosynthetic products allocated to the root system. This shift in resource distribution likely reduces the metabolic demand and oxygen consumption of roots under hypoxic stress, thereby temporarily alleviating oxygen deficiency in the root zone (Zhao et al., 2024). At the same time, increased resource allocation to aboveground tissues helps sustain leaf expansion and photosynthetic function, maintains carbon assimilation capacity, and prepares the plant for rapid recovery once the stress is relieved (Sanchez et al., 2024). Furthermore, both ‘Mahan’ and ‘Pawnee’ cultivars reached their maximum TB and RSR under HO treatment (**Figure 4**), which aligns with previous oxygenation studies on young peach trees conducted by Xiao et al. (2014). This consistency confirms that oxygen enrichment effectively promotes overall dry matter accumulation in woody plants (Zheng et al., 2017). Mechanistically, improved oxygen availability enhances organic matter accumulation through two synergistic pathways. First, it boosts root vitality and aerobic respiration efficiency (Li et al., 2024; Ayi et al., 2019), thereby improving the plant’s capacity for water and nutrient uptake. Second, it improves leaf photosynthetic performance, which facilitates the synthesis and translocation of photosynthetic products to various organs (Sun et al., 2008).

### 4.3 Physiology

Flooding and anoxic conditions disrupt the equilibrium between reactive oxygen species (ROS) production and scavenging in plants, thereby inducing peroxidative damage to cellular membranes. Malondialdehyde (MDA), a product of membrane peroxidation, significantly increases under anoxic conditions, causing structural and functional damage to plant cell membranes (Yu et al., 2015; Bai et al., 2024). Zhao et al. (2023) reported substantial MDA accumulation in *Chionanthus virginicus* leaves after 35 days of flooding treatments, respectively. This accelerated membrane peroxidation, leading to structural disruption and extensive seedling mortality. In this study, MDA content in both cultivars showed a continuous increase throughout the experimental period under HO and LO treatments, reaching peak levels by day 60 (**Figure 5a**). This progressive accumulation indicates severe damage of cell membrane structure during the late stages of flooding stress. Notably, the MDA content under HO treatment was significantly lower than under LO treatment on day 15 and day 60 for ‘Mahan’, and on days 15, 30, and 60 for ‘Pawnee’. These results indicate that oxygen enrichment through aeration effectively mitigates membrane lipid peroxidation and reduces damage to the membrane system (Zheng et al., 2019).

The accumulation of soluble protein (SP) content and the elevation activities of superoxide dismutase (SOD) and catalase (CAT) collectively reflect the osmotic regulation and antioxidant defense mechanisms initiated by plants under stress conditions (Li et al., 2021). Under flooding stress, plants synthesize the osmotic regulator SP to maintain a certain turgor pressure, thereby safeguarding fundamental physiological processes such as cell growth, stomatal opening, and photosynthesis, and enhancing overall stress resistance (Wang et al., 2015). Simultaneously, plants effectively scavenge excess ROS and mitigate oxidative damage by elevating antioxidant enzyme activities such as SOD and CAT (Noctor and Foyer, 1998). In this study, SP content peaked at day 60 across most treatments, with the exception of the ‘Pawnee’ cultivar under LO treatment, which reached its maximum at day 45 (**Figure 5b**). Similarly, the SOD and CAT activities in both cultivars under HO and LO treatments also attained their highest levels at the end of the 60-day experimental period (**Figures 5c, d**). This indicates that *C. illinoinensis* seedlings progressively enhance both osmotic regulation and antioxidant capacity during the late flooding stress, forming a synergistic mechanism that collectively mitigates physiological damage induced by flooding (Yan et al., 2019). Notably, after 60 days of flooding, both cultivars exhibited a consistent gradient in SP content and the activities of SOD and CAT, following the order LO > HO > CK (**Figures 5b–d**). This pattern clearly indicates that the activation level of the plant’s antioxidant defense system is positively correlated with the intensity of the flooding stress (Chen et al., 2025).

**Figure 6:**
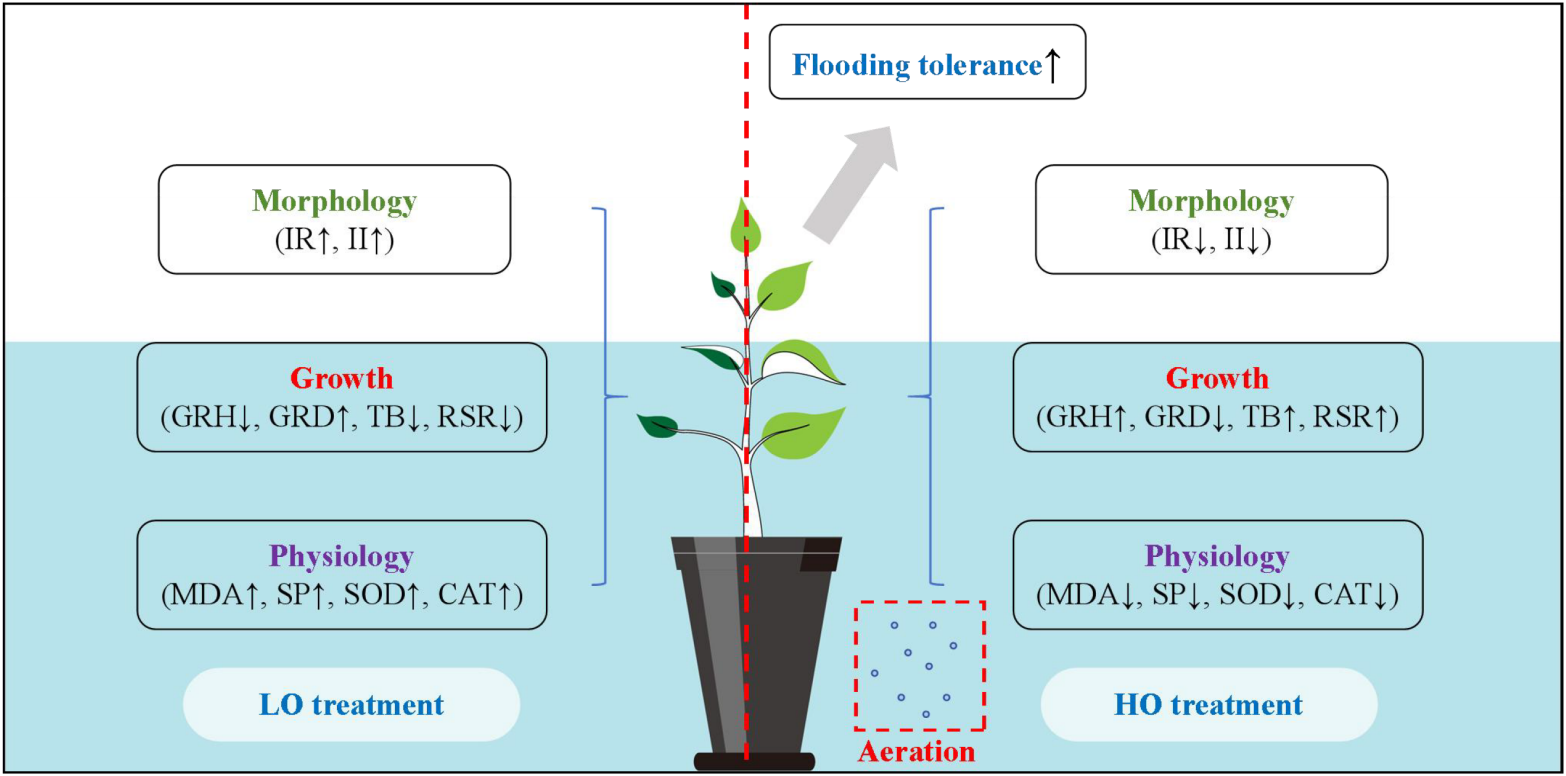
Effects of aeration on morphological, growth, and physiological indicators in plants.

Overall, increasing the DO concentration from 1.88 to 7.11 mg L^-^¹ significantly enhanced the flood tolerance of *C. illinoinensis* (**Table 5**). Specifically, oxygenation reduced leaf damage, decreased key antioxidant enzyme activities, and increased TB accumulation (**Figure 6**). These findings demonstrate that elevated DO is an effective strategy for mitigating flooding stress in plants. This conclusion aligns with previous studies on other mesophytes, including tomato (Li et al., 2025), *Cymbidium* (Song et al., 2024), and rice (Liu et al., 2024). Future research should investigate the effects of different dissolved oxygen gradients on plant flooding tolerance and extend this work to additional mesophytic species. This systematic approach will help elucidate the physiological thresholds and adaptive mechanisms of plants in response to DO fluctuations.

## 5 Conclusion

Under flooding stress, *C. illinoinensis* adapts to hypoxic conditions through a suite of eco-physiological strategies, including suppressed height growth, promoted formation of hypertrophic lenticels at the stem base, accumulation of osmoprotectants, and enhanced antioxidant enzyme activities. Increasing dissolved oxygen in floodwater alleviated stress, reducing the 60-day injury index by 26.43–31.75% and CAT activity by 17.20–23.73% compared to low−oxygen flooding. Although both cultivars exhibit considerable flooding tolerance, ‘Mahan’ demonstrated superior comprehensive stress resistance under high-oxygen treatment (D = 0.56), outperforming ‘Pawnee’ (D = 0.43). Therefore, in flood-prone areas, the simultaneous adoption of water aeration technology and the cultivation of flood-resistant plant cultivars can concurrently enhance plant stress tolerance.

## Data Availability

The raw data supporting the conclusions of this article will be made available by the authors, without undue reservation.
